# Giant Cutaneous Horn of the Cheek: A Case Report

**DOI:** 10.1155/carm/5977603

**Published:** 2026-05-14

**Authors:** Thuc Xuan Nguyen, Khoi Minh Khuat, Hai Thanh Pham

**Affiliations:** ^1^ Faculty of Dentistry, Hai Phong University of Medicine and Pharmacy, Haiphong, Vietnam, hpmu.edu.vn; ^2^ Department of Odonto-Stomatology, Bach Mai Hospital, Hanoi, Vietnam, bachmai.gov.vn

**Keywords:** cutaneous horn, delayed treatment, surgical excision

## Abstract

A cutaneous horn is an uncommon lesion characterized by excessive keratinization, which protrudes above the skin surface and predominantly occurs on sun‐exposed areas. Currently, the exact incidence and prevalence of this disease are not well‐documented in the literature. An 83‐year‐old female patient presented with a cutaneous lesion in the left cheek for more than 15 years. Physical examination revealed the lesion size approximately accounted for 4 cm in length and 1.5 cm in diameter at the base, with a firm, nonerythematous, and painless swelling at its base. The lesion exhibited a distinctive appearance, resembling a large rooster’s spur. Ultrasonographic record identified no definite evidence of deep subcutaneous invasion. To establish a definitive diagnosis, a preoperative biopsy was conducted, which histologically demonstrated a combination of squamous epithelial cells and keratinized debris. Treatment involved local anesthesia and surgical excision therapy. Postoperative management included the administration of antibiotics, NSAIDs for pain relief, and anti‐inflammatory medications. The wound demonstrated favorable healing progress by the first postoperative day. The effective management of cutaneous horn necessitates a comprehensive approach involving precise diagnostic evaluation, proficient surgical excision, and diligent postoperative follow‐up. This case study enhances the current understanding of this uncommon condition and underscores its implications for improving clinical outcomes and patient care.

## 1. Introduction

Cutaneous horn, referred to in Latin as “cornu cutaneum” or colloquially as the devil’s horn, is a conical, hyperkeratotic, protruding lesion that can occasionally grow to a significant size, resembling the appearance of an animal horn [1]. The tumor exhibits a densely compacted keratinized tissue, forming a hardened central core, with multiple cutaneous lesions observed at its basal region [2]. The development of these lesions may be linked to benign, premalignant, or malignant growth processes, particularly in sun‐exposed areas of the skin [3]. Currently, the exact incidence and prevalence of cutaneous horns are not well‐documented in the literature [4].

Clinical examination typically reveals a well‐defined, protruding mass, distinct from surrounding healthy tissue, appearing white or yellow–brown, with a straight or curved structure emerging from the skin. However, variations in shape, color, and size have been documented in some cases [1]. This condition may coexist with other dermatological disorders, including actinic keratosis, sebaceous molluscum, verruca, and malignancies such as squamous cell carcinoma, malignant melanoma, and basal cell carcinoma [5]. Due to its rarity, the prevalence and incidence rates of this disease within the general population remain poorly defined. It is most frequently observed in middle‐aged men, particularly in sun‐exposed regions such as the nose, earlobes, forehead, scalp, and the dorsal aspects of the arms and forearms [6, 7]. Although the lesion primarily causes aesthetic concerns and is typically asymptomatic (lacking pain, itching, or discomfort), it carries a potential risk of malignant transformation. Consequently, histopathological evaluation of the basal lesion is essential to guide appropriate therapeutic interventions [5].

## 2. Case Presentation

**FIGURE 1:**
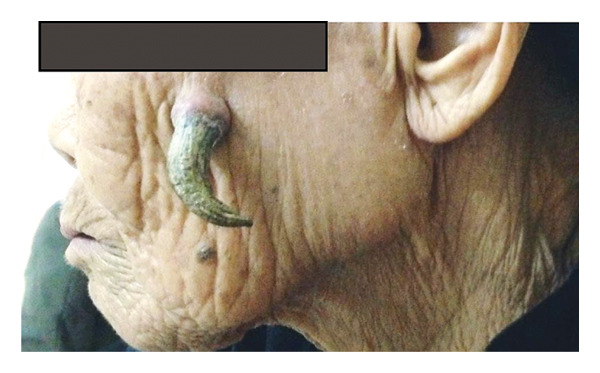
Cutaneous horn on the patient’s left cheek.

An 83‐year‐old female patient, a housewife, was brought to the Department of Odonto‐Stomatology, Bach Mai Hospital, by her niece to be examined for a strange tumor on her left cheek. According to the patient and her family, this strange tumor had been present in the left cheek for more than 15 years. Initially, this lesion was just a raised, hard, smooth mass, about 2‐3 mm in diameter, located on the left cheek, and was asymptomatic upon palpation. Over time, the lesion underwent slow, progressive growth, eventually developing into a sharply curved, horn‐like structure. At the time of presentation, the lesion measured approximately 4 cm in length and 1.5 cm in diameter at the base, with a firm, nonerythematous and painless swelling at its base. The lesion exhibited a distinctive appearance, resembling a large rooster’s spur. Due to social concerns arising from neighbors’ negative remarks, the patient was taken to the hospital for treatment by her family (shown in Figure 1).

Clinical examination did not detect any palpable lymph nodes in the cervical region at the angle of the mandible. The patient reported no prior history of maxillofacial trauma, nor any similar injuries on other parts of the body, and had never undergone surgery. The patient reported that the unusual tumor exhibited no discharge of pus or blood, which led to a delay in seeking medical attention. Although the tumor was not associated with pain or physical discomfort, it significantly impacted the patient’s appearance and daily functioning. The patient experienced difficulty washing her face and was unable to sleep on the left side. Initially, the tumor could be concealed with a mask when going outdoors; however, as it progressively enlarged, complete coverage became unfeasible. This caused the patient to feel increasingly self‐conscious during social interactions (embarrassed by neighbors′ comments). The tumor’s distinctive appearance, resembling a large rooster’s spur, attracted attention and became a subject of discussion among neighbors. Medically, the patient had no history of diabetes but was diagnosed with hypertension, which was not consistently managed.

During the preoperative evaluation, the patient underwent standard diagnostic assessments, including a complete blood count, blood biochemistry panel, and basic coagulation studies, all of which yielded results within normal reference ranges. Ultrasonography identified a superficial hyperechoic lesion measuring approximately 4 cm in length, arising from the epidermal layer with no definite evidence of deep subcutaneous invasion. The underlying soft tissues appeared unremarkable. Differential diagnoses considered by the clinical team included viral verruca and squamous cell carcinoma as potential etiologies for the cutaneous horn‐like lesion. To establish a definitive diagnosis, a preoperative biopsy was conducted, which histologically demonstrated a combination of squamous epithelial cells and keratinized debris (from the original pathology report).

**FIGURE 2:**
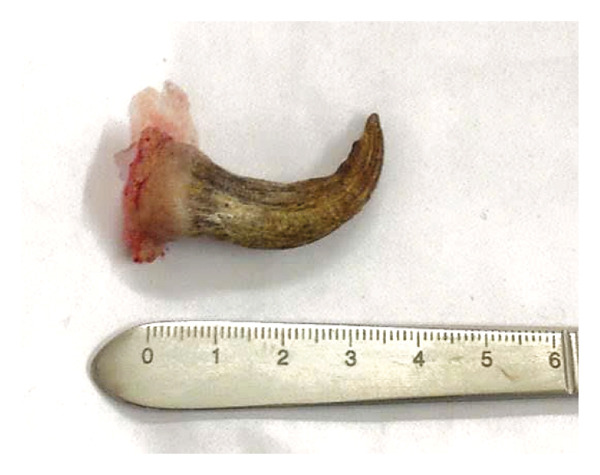
The 4 cm in length cutaneous horn postoperation.

**FIGURE 3:**
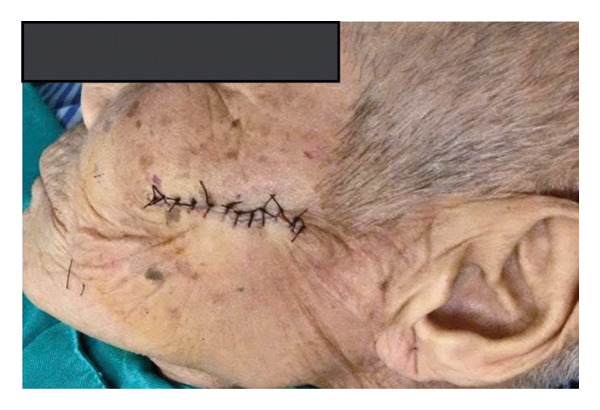
Healing results 1‐day postoperation.

Following local anesthesia administration, the tumor was excised with a scalpel and curved scissors, including a margin of clinically normal‐appearing skin around the base. The excision extended into the subcutaneous tissue to ensure complete removal. Primary closure was achieved with simple interrupted sutures, as illustrated in Figure 2. Postoperative management included oral antibiotics (cephalexin 500 mg twice daily for 5 days), NSAIDs (ibuprofen 400 mg), and topical wound care with saline solution and povidone–iodine twice daily. The wound was treated daily with saline solution and povidone–iodine, demonstrating favorable healing progress by the first postoperative day (shown in Figure 3). The patient expressed satisfaction with the immediate postoperative result. Informed consent for publication of clinical images and case details was obtained from the patient.

## 3. Discussion

The cutaneous horn in this case is conical, curved, with a thick, dark brown keratinized layer on the outside, located on the left cheek. This feature is considered common in cutaneous horn. Although these lesions can manifest on any part of the skin or mucous membranes, they are most frequently reported in areas exposed to sunlight [5]. Supporting this observation, Das et al. documented a clinical case of a cutaneous horn on the abdomen, with the tumor reaching a size of up to 11 cm [8]. A retrospective histopathological analysis of 222 cutaneous horn cases conducted by Mantese et al. revealed that the head was the most common site, accounting for 35.14% of cases [7]. This distribution has been attributed to the association between cutaneous horn development and ultraviolet (UV) light exposure. Consequently, when evaluating patients with cutaneous horns, it is essential to thoroughly assess their occupational history or frequency of outdoor activities [5]. In addition, the presence of pain at the lesion site may indicate an underlying malignant transformation, warranting further investigation.

This case occurred in an 83‐year‐old female patient with a tumor development time of 15 years. This is consistent with the study of Mantese et al., with a higher incidence in women, accounting for 64.86%; the average age of the patients was 67.42 ± 18.36, and the time from onset to recording ranged from 1 week to 20 years [7]. Histopathological analysis of this tumor typically reveals a thickened or hyperkeratotic stratum corneum, which may or may not be accompanied by acanthosis. In benign lesions, parallel or perpendicular arrangements of keratinized tissue layers are commonly observed [9]. The conical portion of the cutaneous horn is predominantly composed of compact keratin, while the base of the lesion represents the region of mitotic activity, which can signify a malignant or premalignant condition. Consequently, obtaining a biopsy from the lesion’s base is essential for precise diagnosis and appropriate therapeutic intervention [3, 6].

The management of cutaneous horn varies according to the underlying etiology of the condition, with three primary treatment approaches: conventional surgical excision, wide excision, medical therapy, or laser ablation [1]. For benign lesions, initial observation may be employed, or surgical excision can be conducted for cosmetic reasons upon patient request. Regular follow‐up is advised to monitor tumor progression, while wide excision is recommended for premalignant or malignant cases [5]. Laser excision or electrocautery may be utilized for smaller lesions, primarily for cosmetic purposes at the patient’s discretion; however, these methods are not considered substitutes for excision biopsy [3]. In cases of confirmed squamous cell carcinoma or basal cell carcinoma, a metastatic evaluation is essential, particularly involving assessment of adjacent lymph nodes and lymph node biopsy [1, 3]. Psychological aspects should also be considered, as patients may experience embarrassment, perceiving the lesion as a hindrance to their daily life and social interactions [10]. This perception often leads to delays in seeking specialized medical care. Clinicians must acknowledge and address these cosmetic concerns through appropriate counseling, as they can profoundly influence the patient’s psychological well‐being [11, 12].

Recent literature continues to expand our understanding of cutaneous horn pathology. Tommasino et al. [13] recently reported a case of cutaneous horn arising from an underlying dermatofibroma, highlighting that even benign mesenchymal tumors can present with this striking clinical appearance. Their observation reinforces the critical importance of histopathological evaluation of the lesion base, as the keratinous projection itself provides no indication of the nature of the underlying process. This is particularly relevant to our case, where detailed histopathological characterization was unfortunately not possible. Second, we have explicitly stated that the lack of long‐term follow‐up represents a significant limitation of this case report. The patient, an 83‐year‐old female from a rural area, was lost to systematic follow‐up after the first postoperative week due to transportation difficulties and family circumstances. We have made multiple attempts to contact the patient and family for updated follow‐up information, but have been unsuccessful. The only follow‐up data available to us are the Day‐1 postoperative assessment documented in the medical records.

## Author Contributions

Thuc Xuan Nguyen: conceptualization, data curation, investigation, methodology, formal analysis, resources, writing–original draft, writing and editing the final manuscript, and project administration.

Khoi Minh Khuat: data curation and writing–original draft.

Hai Thanh Pham: conceptualization, data curation, investigation, methodology, formal analysis, resources, writing–original draft, writing and editing the final manuscript, and project administration.

## Funding

This study was not supported by any sponsor or funder.

## Disclosure

All authors have reviewed and approved the final version of this manuscript for submission.

## Ethics Statement

Ethical approval is not required for this study in accordance with local or national guidelines.

## Consent

The patient signed an informed consent form to allow the publication of this case report and accompanying images.

## Conflicts of Interest

The authors declare no conflicts of interest.

## Data Availability

The data that support the findings of this study are not publicly available due to privacy reasons but are available from the corresponding author, upon reasonable request.
